# Meta-analysis of association between Arg326Gln (rs1503185) and Gln276Pro (rs1566734) polymorphisms of *PTPRJ* gene and cancer risk

**DOI:** 10.1007/s13353-019-00481-3

**Published:** 2019-01-19

**Authors:** Izabela Laczmanska, Maria M. Sasiadek

**Affiliations:** Genetics Department, Wroclaw Medical University, Marcinkowskiego 1, 50-368, Wroclaw, Poland

**Keywords:** *PTPRJ*, SNP, Cancer, Meta-analysis

## Abstract

Protein tyrosine phosphatase receptor type J (*PTPRJ*, *DEP1*) is a tumour suppressor gene that negatively regulates such processes as angiogenesis, cell proliferation and migration and is one of the genes important for tumour development. Similar to other phosphatase genes, PTPRJ is also described as an oncogene. Among various genetic changes characteristic for this gene, single nucleotide polymorphisms (SNPs) constituting benign genetic variants that can modulate its function have been described. We focused on Gln276Pro and Arg326Gln missense polymorphisms and performed a meta-analysis using data from 2930 and 852 patients for Gln276Pro and Arg326Gln respectively in different cancers. A meta-analysis was performed based on five articles accessed via the PubMed and Research Gate databases. Our meta-analysis revealed that for Arg326Gln, the presence of the Arg (C) allele was associated with lower risk of some cancers, the strongest association was observed for colorectal cancer patients, and there was no association between Gln276Pro (G>T) polymorphism and cancer risk. The polymorphisms Arg326Gln and Gln276Pro of the *PTPRJ* gene are not associated with an increased risk of cancer except for the Arg326Gln polymorphism in colorectal cancer. Large-scale studies should be performed to verify the impact of this SNP on individual susceptibility to colorectal cancer for given individuals.

## Introduction

Protein tyrosine phosphatase receptor type J (*PTPRJ*, *DEP1*) is described as a tumour suppressor gene that negatively regulates angiogenesis, cell proliferation and migration and therefore is involved in tumour progression in some human cancers (Aya-Bonilla et al. 2013; Bilotta et al. 2016; Fournier et al. 2016; Zhang et al. 2017). The proven impact of PTPRJ on cellular biology results from its role in dephosphorylation of some kinases such as PDGFR, EGFR, VEGFR2, HGFR, PI3K (p85), MAPK (ERK1/2), FLT3 and RET and also proteins involved in cell adhesion such as c-Src, p120-catenin, VE-cadherin, THBS1 and ZO-1 (Aya-Bonilla et al. 2013; Fournier et al. 2016; Qiao et al. 2016; Steinman et al. 2016). Although *PTPRJ* is mainly classified as a tumour suppressor gene, it has also been described as an oncogene. *PTPRJ* loss of heterozygosity (LOH) has been reported in several cancers such as colon, breast, thyroid, meningioma, non-Hodgkin lymphoma and cervical carcinoma (Ruivenkamp et al. 2003; Iuliano et al. 2010; Aya-Bonilla et al. 2013; Yan et al. 2015). Moreover, germline epigenetic silencing of this gene in early-onset familiar colorectal cancer (Venkatachalam et al. 2010) as well as the loss of *PTPRJ* expression in association with an advanced tumour stage and poor differentiation in oesophageal squamous cell carcinoma was described (Qiao et al. 2016). In contrast, the role of PTPRJ in VEGF-dependent Src activation, and therefore capillary formation and permeability, has been demonstrated in both a mouse model and human breast cancer (Spring et al. 2015; Fournier et al. 2016). Also a soluble short isoform, sPTPRJ, secreted into endothelial and tumour cells was shown to down-regulate endothelial adhesion molecules and to promote angiogenesis in glioblastoma (Bilotta et al. 2016). It was also reported that PTPRJ agonist nonapeptide led to reduction of cell proliferation and promotion of apoptosis in cancer cell lines and also inhibited tube formation in an in vitro experiment (Ortuso et al. 2013). These findings confirmed the hypothesis that protein tyrosine phosphatases via dephosphorylation play a dual role in carcinogenesis (as tumour suppressors and oncogenes) as they are crucial regulators of the activity of a variety of proteins (Julien et al. 2011; Zhao et al. 2015).

As has been demonstrated, *PTPRJ* is expressed in various types of cells (e.g. epithelial, haematopoietic and endothelial cells and many cancer cell lines), and thus its effect on different cancer types seems to be indisputable (Fournier et al. 2016).

Single nucleotide polymorphisms (SNPs) are benign genetic variants that can modulate expression, folding, activity, binding affinity and other protein functions and therefore are intensively studied in many different diseases (Katsonis et al. 2014). The Gln276Pro (rs1566734) and Arg326Gln (rs1503185) polymorphisms in *PTPRJ* are missense SNPs that were previously genotyped in colorectal, thyroid, lung, head and neck, oesophageal and breast cancers (Iuliano et al. 2004, 2010; Toland et al. 2008; Mita et al. 2010; Wei et al. 2013; Shangkuan et al. 2017). Both polymorphisms are located in the extracellular region of PTPRJ and are involved in fibronectin-like type III domain formation (Ruivenkamp et al., 2002).

In our study, we investigated whether Gln276Pro and Arg326Gln missense polymorphisms are risk factors in various cancers. Because a meta-analysis is a proper tool for evaluating the association between gene polymorphisms and cancer risk, we analysed data collected from all five studies published to date focusing on these SNPs (*n* = 2930 and *n* = 852 for Gln276Pro and Arg326Gln respectively) in different cancers.

## Materials and methods

### Meta-analysis—selection of studies

We searched PubMed and ResearchGate databases to compare our investigation with research on other populations by collecting articles published until December 2017 using the following terms: “PTPRJ[All Fields] OR “DEP-1[All Fields] AND (“neoplasms“[MeSH Terms] OR “neoplasms“[All Fields] OR “cancer”[All Fields])”. Reference lists and conference reports were included in the analysis. Finally, 82 publications which were focused on comparison between cancer patients and a healthy control group were found. Review papers, meta-analyses and papers describing other PTPRJ gene polymorphisms were excluded. After applying the above restriction, five papers that compared the frequency of Arg326Gln and Gln276Pro of the *PTPRJ* gene in cancer patients and healthy controls were included in our final meta-analysis. Odds ratios (OR), together with 95% confidence intervals (CIs), were used to assess the strength of associations between polymorphisms of *PTPRJ* and the risk of cancer. The search strategy is reported according to the PRISMA (http://www.prisma-statement.org/) reporting guidelines.

### Meta-analysis—statistical analysis

The number of alleles was calculated according to the following formula: NA = *n* × *q*, where *n* is the total number of genotypes in the group, and *q* is the probability of the allele (R and Q for both polymorphisms) (Łaczmański et al. 2015).

The fixed-effects model and the DerSimonian-Laird random-effects model (with weights based on the inverse variance) were used to calculate summary odds ratios (ORs), and both within- and between-study variations were considered. Variants of this model were considered both within our study and for other studies. The significance level was set at 5% and random-effects analysis was selected. All statistical analyses were performed using Statistica ver. 10 software (StatSoft, USA) with the add-on Medical Package.

## Results

Five articles describing the association between *PTPRJ* Arg326Gln and Gln276Pro polymorphisms in six different cancers (breast, colorectal, oesophagus, head and neck, lung and thyroid) were included in the analysis. In total, for the Arg326Gln polymorphism, 852 cases and 1071 controls while, for the Gln276Pro polymorphism, 2930 cases and 3408 controls were included in the pooled analyses (Tables 1, 2).

**Table 1:** Characteristics of studies included in the meta-analysis of the SNP at C>T, Arg326Gln (rs1503185) of *PTPRJ*

Study	Area	Race	Type of cancer	No. of cases	No. of controls	Genotypes in case group *n* (%)			Genotypes in controls *n* (%)		
						Arg326Gln (R326Q) (rs1503185)			Arg326Gln (R326Q) (rs1503185)		
						RR	RQ	QQ	RR	RQ	QQ
Iuliano et al. 2004	Italy	Caucasian	TA TC	22 66	54 54	11 45	10 17	1 4	32 32	20 20	2 2
Mita et al. 2010	Japan	Asian	LAD LSQ HNSCC CRC ESCC	108 59 92 113 193	819 819 819 819 819	66 36 58 51 106	34 17 27 45 77	8 6 7 17 10	510 510 510 510 510	272 272 272 272 272	37 37 37 37 37
Wei et al. 2013	China	Japan	BC	199	198	104	75	20	87	92	19

*TA* thyroid adenoma, *TC* thyroid carcinoma, *CRC* colorectal cancer, *LAD* lung adenocarcinoma, *LSQ* lung squamous cell carcinoma, *HNSCC* head and neck squamous cell carcinoma, *ESCC* oesophageal cancer, *BC* breast cancer

**Table 2:** leftacteristics of studies included in the meta-analysis of the SNP at G>T, Gln276Pro (rs1566734) of *PTPRJ*

Study	Area	Race	Type of cancer	No. of cases	No. of controls	Genotypes in case group *n* (%)			Genotypes in controls *n* (%)		
						Gln276Pro (Q276P) (rs1566734)			Gln276Pro (Q276P) (rs1566734)		
						QQ	QP	PP	QQ	QP	PP
Iuliano et al. 2004	Italy	Caucasian	TA	22	126	11	10	1	32	20	74
			TC	66	54	45	17	4	32	20	2
Puijenbroek et al. 2005	The Netherlands	Caucasian	CRC	222	156	149	64	9	103	47	6
Toland et al. 2008	Israel	Mostly of Ashkenazi Jewish ancestry	CRC	1897	1954	1047	701	149	1094	717	143
Mita et al. 2010	Japan	Asian	LAD LSQ HNSCC CRC ESCC	108 59 92 113 193	814 814 814 814 814	66 36 58 51 106	34 17 27 45 77	8 6 7 17 10	499 499 499 499 499	281 281 281 281 281	34 34 34 34 34
Iuliano et al. 2010	Finland France Italy	Caucasian	TC	156	299	111	36	9	197	91	11

*TA* thyroid adenoma, *TC* thyroid carcinoma, *CRC* colorectal cancer, *LAD* lung adenocarcinoma, *LSQ* lung squamous cell carcinoma, *HNSCC* head and neck squamous cell carcinoma, *ESCC* oesophageal cancer, *BC* breast cancer

### Arg326Gln

**Fig. 1:**
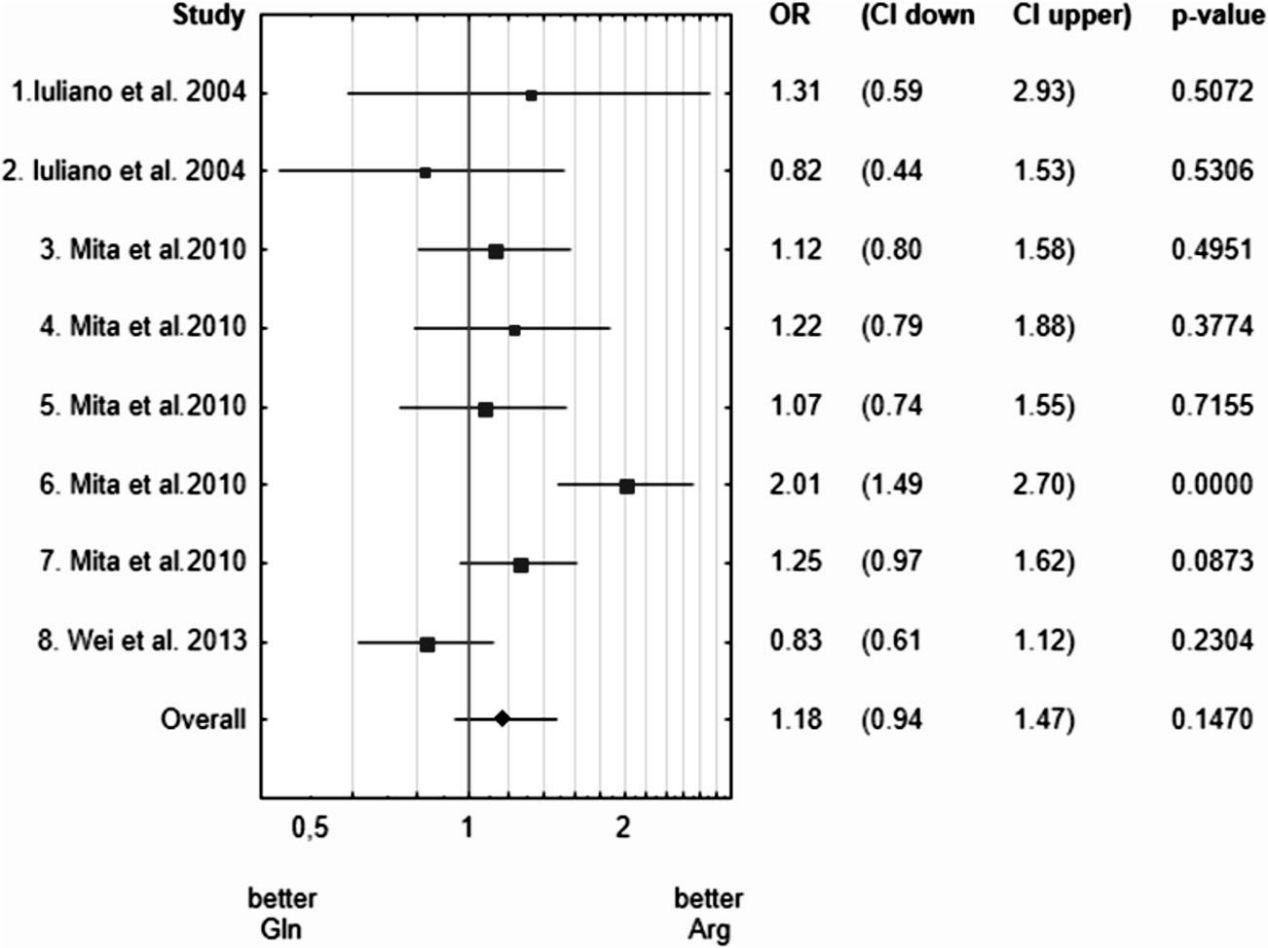
A Forest plot for meta-analysis of association between Arg326Gln *PTPRJ* polymorphism and cancer risk. 1, Thyroid adenomas; 2, Thyroid carcinomas; 3, LAD; 4, LSQ; 5, HNSCC; 6, CRC; 7, ESCC; 8, BC

The presence of the Arg (C) allele is associated with lower risk of different cancers. The strongest association was observed for colorectal cancer patients (Fig. 1).

### Gln276Pro

**Fig. 2:**
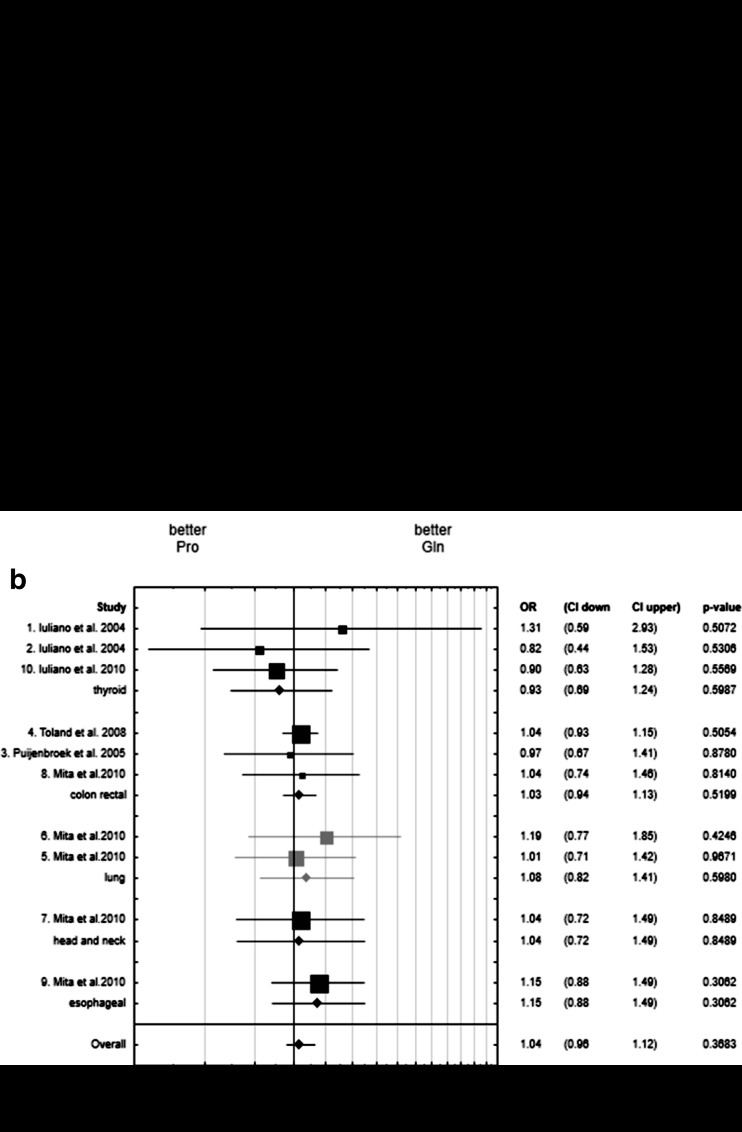
**a** Forest plot for meta-analysis of association between Gln276Pro *PTPRJ* polymorphism and cancer risk. 1, Thyroid adenomas; 2, Thyroid carcinomas; 3, CRC; 4, CRC; 5, LAD; 6, LSQ; 7, HNSCC; 8, CRC; 9, ESCC; 10, Thyroid carcinomas. **b** Forest plot for meta-analysis of association between Gln276Pro *PTPRJ* polymorphism and cancer risk in different sub-groups of cancers

There was no association between Gln276Pro (G>T) polymorphism and cancer risk (Fig. 2).

## Discussion

The dual role of protein phosphatases makes them one of the most important gene groups (together with kinase genes) in cancer development and progression. Many studies on protein tyrosine phosphatase alterations at epigenetic and genetic levels confirm that loss of their function is a characteristic feature for cancer cells. Beside genetic alterations such as loss of heterozygosity, point mutations and aberrant methylation, also single nucleotide polymorphisms in protein tyrosine phosphatase genes are suspected to be responsible for variable risk of cancers in patients.

Due to the fact that data from many studies on different types of cancers are nowadays easily available, the meta-analysis of a huge number of results is possible. The strength of meta-analysis lies in its ability to collect together all published data from different laboratories for different ethnic groups of patients. In our meta-analysis, to our best knowledge, we analysed all published data for *PTPRJ* Arg326Gln and Gln276Pro polymorphisms in different cancers (breast, colorectal, oesophagus, head and neck, lung and thyroid cancer).

Our meta-analysis revealed that:

- For Arg326Gln, the presence of the Arg (C) allele was associated with lower risk of some cancers and the strongest association was observed for colorectal cancer patients; however, the data for CRC were obtained only from one study (Mita et al. 2010).
  The association for Arg326Gln we revealed was strongly influenced by data for 113 patients with CRC from Mita et al. 2010. When these data were excluded from the analysis there was no association of Arg326Gln and cancer risk (*p* value = 0.2959).
- There was no association between Gln276Pro (G>T) polymorphism and cancer risk.

Cancer development and progression are complex processes, and, except for major impact genes, there is usually no simple answer as to which moderate and especially minor impact genes are important for them. Genetic polymorphisms (mainly SNPs) have been widely studied for many years, but although many experiments show the statistical significance of these variables, their effect is always dependent on other genetic and environmental factors and cannot be precisely defined. A meta-analysis of all available data can show which genetic changes are important and which may be excluded from further studies.

Both polymorphisms we studied are missense SNPs, and the changed amino acids are located in the extracellular region of PTPRJ involved in fibronectin-like type III domain formation. Because the role of *PTPRJ* in cancers has been widely described and many studies confirm its role in this process, we may suppose that the location of Gln276Pro (rs1566734) and Arg326Gln (rs1503185) polymorphisms in *PTPRJ* or amino acid changes and what they underlie are not important for the role of *PTPRJ* in carcinogenesis. Nevertheless, the association of the presence of the Arg (C) allele for the Arg326Gln polymorphism with lower risk especially for colorectal cancer seems to be an interesting subject for further studies to finally confirm or not the results of Mita et al. (2010).

Some limitations of our meta-analysis should be pointed out, such as the low number of studies included in the analysis (only five publications) with only 852 cases and 1071 controls for Arg326Gln and 2930 cases and 3408 controls for the Gln276Pro polymorphism. The ethnicity of patients was also different, for the Arg326Gln polymorphism the majority of patients were Asians, while for Gln276Pro the biggest group had Ashkenazi Jewish ancestry, and the second in number was Asian. Despite these considerations, our results were surprisingly consistent for all analysed cancer sub-types. Another limitation of our paper is that the meta-analysis included one study with 5 analyses of different cancer patients groups for which there was only a single control group. However, the control group consisted of more than 800 healthy individuals (Mita et al. 2010). In our opinion, one group of healthy individuals as a control group for 5 different cancer groups is permissible, because the segregation of polymorphic alleles in healthy individuals should be in Hardy-Weinberg equilibrium.

## Conclusion

The polymorphisms Arg326Gln and Gln276Pro of the *PTPRJ* gene are not associated with an increased risk of cancer except for the Arg326Gln polymorphism in colorectal cancer. Thus, further large-scale studies should be performed to verify the impact of this SNP on individual susceptibility to colorectal cancer for given individuals.
